# Chasing Digital Perfection in a Filtered World: The Role of Dermatologists in Addressing Social Media Dysmorphia

**DOI:** 10.1111/jocd.70672

**Published:** 2026-01-15

**Authors:** Kelly Frasier, Nicole Werpachowski, Mary Grace Hash, Raman Madan

**Affiliations:** ^1^ Department of Dermatology Northwell New Hyde Park New York USA; ^2^ Department of Medicine Lenox Hill Hospital, Northwell Health New York New York USA; ^3^ Edward via College of Osteopathic Medicine Auburn Alabama USA; ^4^ Department of Dermatology, Northwell New Hyde Park New York USA

**Keywords:** cosmetic dermatology, digital beauty standards, digital literacy, psychodermatology, social media dysmorphia

## 1 Introduction

The rise of visually driven platforms like TikTok, Instagram, and Snapchat has reshaped how people perceive and present themselves, making appearance central to digital identity. Filters and editing tools allow users to alter skin, facial symmetry, and proportions, normalizing idealized, unattainable standards. This curated reality deeply impacts self‐esteem, particularly among adolescents and young adults who rely on social validation. As these dynamics intensify, dermatologists must manage both cosmetic demands and the psychological toll of a growing phenomenon often described as social media dysmorphia. Understanding this intersection of digital culture, mental health, and cosmetic dermatology reveals a timely opportunity to redefine patient care in the age of virtual beauty.

## 2 Social Media Dysmorphia: A Psychosocial Syndrome

Social media dysmorphia describes body‐image disturbances that emerge from external digital influences shaped by filters, editing tools, and social comparisons rather than internalized flaws [1, 2]. A cross‐sectional study of 740 adolescents found that over 4 h of daily social media use correlated with significantly higher Dysmorphic Concern Questionnaire scores [2], particularly on image‐based platforms like Instagram. Social media dysmorphia is more accurately represented as a situational variant of dysmorphic concern that overlaps substantially with body dysmorphia disorder (BDD).

Augmented reality tools promote unrealistic features: flawless skin, symmetrical faces, Eurocentric traits. These edits set unattainable standards and reinforce self‐doubt. “Snapchat dysmorphia” describes patients who bring filtered selfies to dermatologists, hoping to replicate them [3]. “Zoom dysmorphia” emerged during the COVID‐19 pandemic, as prolonged video calls drove rising cosmetic consultations based on distorted self‐perception [4]. This highlights how digital platform‐specific features can serve as modern triggers for dysmorphic symptoms and amplify appearance dissatisfaction.

At the core is a misalignment between the unedited self and the idealized online persona. The psychological conflict that emerges from this disconnect drives persistent dissatisfaction and amplifies the desire for physical alterations. However, it remains best conceptualized within the broader framework of BDD, reflecting a broader cultural transformation in appearance standards.

## 3 Psychological Consequences and the Cosmetic Feedback Loop

Constant exposure to curated content distorts self‐image. Adolescents, in formative stages of identity, are especially vulnerable. Studies show that reducing social media use can significantly improve body image in teens and young adults [5]. Filters deepen this dependency by framing unedited appearances as flawed, encouraging repeated compulsive use of digital tools to boost self‐esteem.

This feedback loop consisting of comparison, distress, and platform re‐engagement often leads to cosmetic intervention. Users spending 4 to 7 h daily on visual platforms are more likely to report BDD symptoms and pursue aesthetic procedures. Yet these interventions rarely resolve underlying dissatisfaction. Among BDD patients, 38% undergo rhinoplasty, 8.2% breast augmentation, and over 50% receive collagen injections; yet 75% remain unsatisfied, and 16% report worsening symptoms [1]. The gap between expected and actual outcomes can escalate emotional distress and repeat procedures. Sustainable solutions require addressing psychological roots through education, therapy, and self‐acceptance, rather than focusing solely on aesthetic correction.

## 4 Dermatologic Implications and Ethical Considerations

Dermatologists are often the first clinicians to encounter individuals shaped by social media dysmorphia. Among BDD patients, dermatologic and cosmetic procedures are the most frequently pursued treatments [1]. Many cite filtered selfies or influencer images as motivations for seeking fillers, neuromodulators, or skin resurfacing. These consultations often come with unrealistic expectations.

Physicians must balance empathy with ethical responsibility. When filtered ideals replace realistic outcomes, cosmetic procedures risk exacerbating distress. Patients with suspected BDD or dysmorphia should be screened and referred to mental health professionals. Clear referral triggers include the presence of suicidal ideation, significant functional impairment, degree of conviction about perceived flaws, and patients who demonstrate procedural fixation (e.g., repeatedly seeking cosmetic interventions). Many lack insight into the psychological drivers behind their requests, compromising informed consent. Early identification and a referral‐based care model reduce patient harm and legal risk. This approach also supports ethical practice grounded in non‐maleficence. Tools like motivational interviewing and augmented reality can help manage expectations and support sound decision‐making.

Dermatologists must also consider their own digital presence. While social media offers education and visibility, promotional content can unintentionally reinforce harmful norms. Using unedited visuals, diverse representations, and disclaimers fosters authenticity and trust. Some practical standards for professional social media use include disclosure of lighting or editing adjustments applied to images and avoiding filtered or digitally enhanced before‐and‐after images that can potentially misrepresent clinical outcomes. Dermatologists should also clearly label sponsored content, distinguishing evidence‐based educational posts from marketing‐driven recommendations. Without such disclosure, online promotional content risks being perceived as medical advice by patients. Rather than contributing to the problem, dermatologists can counteract dysmorphia by promoting realism, transparency, and empowerment.

## 5 Interdisciplinary Strategies: Integrating Dermatology and Mental Health

Addressing social media dysmorphia requires cross‐disciplinary strategies. Dermatologists should integrate psychological screenings into consults, especially with adolescents or those expressing dissatisfaction. Collaborative care models involving mental health professionals enable dual interventions, treating both physical concerns and emotional distress. Cognitive behavioral therapy (CBT) and motivational interviewing are effective in challenging negative self‐perceptions [6]. When combined with ethical dermatologic treatment, these approaches offer comprehensive support.

Public health campaigns must also play a role. Adolescents should be taught to critically evaluate online content, recognizing the prevalence of digitally altered images. Digital literacy programs can reduce adolescents' susceptibility to appearance‐based distress. These programs educate young users on the prevalence of altered imagery and unrealistic standards. Body‐positive messaging that celebrates diverse appearances can reshape beauty norms. Campaigns should consider cultural and gender differences, ensuring inclusive outreach. At the policy level, regulations mandating disclosure of filters in advertisements or influencer promotions may reduce harm by promoting transparency and consumer protection.

## 6 The Influencer Effect: Authenticity vs. Aspiration

Influencers are major drivers of social media dysmorphia. Their curated, filtered, and often enhanced content sets unrealistic standards and pressures followers to emulate idealized appearances. Unlike traditional advertising, influencer content is seen as authentic, making it more persuasive. Cosmetic trends often follow influencer promotion. For instance, widely circulated online discussions about “Kylie Jenner lip filler” and related search activity in July 2018 mirrored her Instagram activity, although these observations remain largely anecdotal in nature with uncertain clinical implications [7]. Similarly, practices like “mewing” have gained popularity across social media despite limited scientific evidence supporting their efficacy, amplified by influencer reach [7].

These trends shape beauty norms, especially among adolescents, who are more susceptible to internalizing ideals. Influencer‐driven content frames cosmetic enhancement as routine self‐care rather than medical intervention, reinforcing appearance‐based worth. Addressing this influence requires structural changes, including platform accountability, mandatory disclosures, and content regulation. Dermatologists can counteract misinformation by using their platforms to promote evidence‐based messaging, highlight realistic outcomes, and challenge misleading narratives.

## 7 Future Research and Clinical Direction

As social media continues to evolve, research must assess its long‐term effects on mental health and dermatologic practice. Longitudinal studies can illuminate how social media dysmorphia influences patient satisfaction, procedure outcomes, and emotional well‐being. Trials exploring the integration of CBT with minimally invasive procedures may provide new models for treating psychosocial and aesthetic concerns concurrently. Understanding how sociocultural and algorithmic forces shape perceptions of beauty will be key to designing effective interventions.

Dermatology must expand its scope, engaging with the broader forces that shape self‐image. Through collaboration with psychologists, public health experts, and digital platforms promoting ethical social media engagement, dermatologists can serve as agents of change in restoring authenticity and self‐acceptance to modern beauty standards.

## 8 Conclusion

Social media dysmorphia is a digitally mediated crisis of self‐image, fueled by curated content, algorithmic validation, and culturally reinforced beauty ideals. Dermatologists are uniquely positioned to address its psychological and aesthetic consequences by bridging clinical care with ethical advocacy. Through transparent consultations, interdisciplinary collaboration, and responsible social media practices, they can counteract harmful narratives and empower patients to embrace diverse, realistic expressions of beauty. In doing so, dermatology not only treats the visible consequences of dysmorphia but also redefines its role in cultivating self‐worth in the digital age.

## Author Contributions

Kelly Frasier conceptualized and drafted the manuscript. All authors wrote a section of the manuscript. All authors provided critical revisions and contributed to the interpretation of evidence related to this manuscript. All authors read and approved the final manuscript.

## Ethics Statement

The authors have nothing to report.

## Conflicts of Interest

The authors declare no conflicts of interest.

## Data Availability

The authors have nothing to report.
